# African swine fever virus QP383R dampens type I interferon production by promoting cGAS palmitoylation

**DOI:** 10.3389/fimmu.2023.1186916

**Published:** 2023-05-09

**Authors:** Siyuan Hao, Xiaojie Zheng, Yingqi Zhu, Yao Yao, Sihan Li, Yangyang Xu, Wen-hai Feng

**Affiliations:** ^1^ State Key Laboratory of Animal Biotech Breeding, College of Biological Sciences, China Agricultural University, Beijing, China; ^2^ Frontiers Science Center for Molecular Design Breeding, College of Biological Sciences, China Agricultural University, Beijing, China; ^3^ Ministry of Agriculture Key Laboratory of Soil Microbiology, College of Biological Sciences, China Agricultural University, Beijing, China; ^4^ Department of Microbiology and Immunology, College of Biological Sciences, China Agricultural University, Beijing, China

**Keywords:** ASFV, QP383R, type I interferons, cGAS, palmitoylation

## Abstract

Cyclic GMP-AMP synthase (cGAS) recognizes viral DNA and synthesizes cyclic GMP-AMP (cGAMP), which activates stimulator of interferon genes (STING/MITA) and downstream mediators to elicit an innate immune response. African swine fever virus (ASFV) proteins can antagonize host immune responses to promote its infection. Here, we identified ASFV protein QP383R as an inhibitor of cGAS. Specifically, we found that overexpression of QP383R suppressed type I interferons (IFNs) activation stimulated by dsDNA and cGAS/STING, resulting in decreased transcription of IFNβ and downstream proinflammatory cytokines. In addition, we showed that QP383R interacted directly with cGAS and promoted cGAS palmitoylation. Moreover, we demonstrated that QP383R suppressed DNA binding and cGAS dimerization, thus inhibiting cGAS enzymatic functions and reducing cGAMP production. Finally, the truncation mutation analysis indicated that the 284-383aa of QP383R inhibited IFNβ production. Considering these results collectively, we conclude that QP383R can antagonize host innate immune response to ASFV by targeting the core component cGAS in cGAS-STING signaling pathways, an important viral strategy to evade this innate immune sensor.

## 1 Introduction

African swine fever virus (ASFV) is a large double-stranded, cytoplasmic DNA arbovirus belonging to the genus *Asfivirus* in the family *Asfarviridae* (1, 2). The genomic size of ASFV is approximately 170 to 193 kb, and the genome encodes more than 150 viral proteins that play important roles in viral assembly, viral replication, virus-host interaction, and immune evasion. However, many viral proteins have unknown functions (3–5). ASFV replicates mainly in the cytoplasm of monocyte- and macrophage-lineage cells (6). As a complex enveloped DNA virus, ASFV is responsible for African swine fever disease (ASF). And this highly contagious hemorrhagic viral disease in domestic pigs (*Sus scrofa domestica*) and wild boars (*Sus scrofa*) has a morbidity and mortality rate of up to 100% and threatens the global pork supply and food security (7). Despite extensive research, there are no effective vaccines or antiviral drugs commercially available for the prevention and control of this deadly disease. Depletion of the virulence factors from field viruses to generate live-attenuated vaccines (LAVs) is the most promising strategy for the development of efficient vaccines so far (8). Therefore, it is critical to identify the virulence and immunosuppressive factors to provide potential targets for vaccine design.

The innate immune system is the first line of host defense against invading pathogens. Upon pathogens infection, cellular pattern recognition receptors (PRRs) recognize pathogen-associated molecular patterns (PAMPs) (9–11), which triggers a series of signaling events that lead to the induction of type I interferons (IFNs) (12), proinflammatory cytokines and other downstream effectors (13, 14). These effectors mediate the inhibition of microbial replication, clearance of infected cells and facilitation of adaptive immune response to eliminate infected pathogens (15, 16).

Among PRRs, cyclic GMP-AMP synthase (cGAS) is a recently identified DNA sensor, which plays a pivotal role in recognizing cytosolic DNA (17). Mechanistically, after binding to dsDNA, cGAS forms a 2:2 complex with DNA, which allows the rearrangement of the cGAS catalytic pocket for the subsequent binding, and then catalyzes its substrates ATP and GTP to produce a cyclic dinucleotide: 2’-3’-cGAMP. As a cytosolic second messenger, cGAMP binds to the adaptor protein STING, and causes a 180° rotation of its carboxyl ligand-binding domain relative to its transmembrane domain, leading to STING activation (18–20). Activated STING serves as the platform for recruitment and activation of TBK1, which in turn phosphorylates STING and IRF3. Phosphorylated IRF3 forms a homo-dimer to enter the nucleus, leading to the transcription of type I IFNs and other antiviral effector genes (21–23). In contrast, STING activation stimulates the inhibitor of nuclear factor-κB (IκB) kinase to release NF-κB, which translocates to the cell nucleus and activates the transcription of type I IFNs and proinflammatory cytokine-related genes (24).

QP383R is classified as an uncharacterized protein, which consists of 383 amino acids. Recently, it has been reported that QP383R represses inflammatory responses by inhibiting AIM2 inflammasome activation (25). In our study, we identified ASFV QP383R as a negative regulator of cGAS-STING mediated innate immunity. We found that overexpression of QP383R reduced dsDNA-triggered and cGAS-STING-mediated innate antiviral response. Furthermore, we found that QP383R interacted with the nucleotidyltransferase (NTase) domain of cGAS through its C-terminal tail (aa284-383). Palmitoylation is an important post-translational modification of cGAS, which restricts its enzymatic activity in the presence of dsDNA. We showed that QP383R promoted cGAS palmitoylation, and also impeded the DNA binding ability and dimerization of cGAS. Importantly, QP383R inhibited cGAS enzymatic functions and reduced cGAMP production, thereby attenuating the downstream innate immune response. Together, our findings reveal a novel immune evasion mechanism of ASFV mediated by the QP383R protein, implying that the QP383R gene could be used as a candidate target gene for the ASFV live-attenuated vaccines.

## 2 Results

### 2.1 QP383R inhibits cGAS-STING-mediated signaling

**Figure 1:**
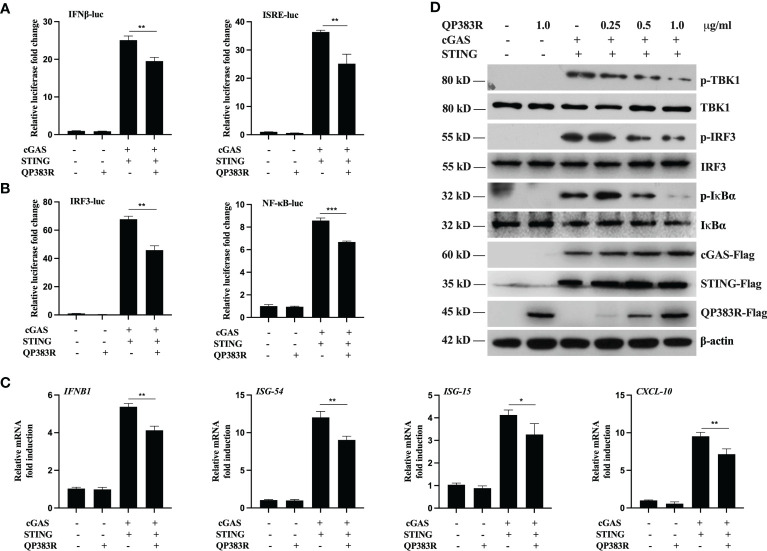
QP383R is an inhibitor of cGAS-STING-mediated signaling. **(A, B)** HEK239T cells were co-transfected with cGAS-Flag (40 ng/ml), STING-Flag (160 ng/ml), QP383R-Flag (0.5 µg/ml) or their empty vectors, and the indicated reporters (50 ng/ml) and pRL-TK (8 ng/ml). Twenty-four hours later, the cells were harvested to determine the activities of IFNβ and ISRE promoters **(A)**, and IRF3 and NF-κB promoters **(B)** by luciferase assays. **(C)** HEK239T cells were co-transfected with cGAS-Flag (40 ng/ml), STING-Flag (160 ng/ml), QP383R-Flag plasmid (0.5 µg/ml) or their empty vectors for 24 h. The expression of antiviral genes including *IFNB1*, *ISG54*, *ISG15*, and *CXCL10* were examined by qPCR. **(D)** HEK293T cells were transfected with cGAS-Flag and STING-Flag or an empty vector, and the indicated amounts of QP383R-Flag plasmids for 24 h. Immunoblots were performed with anti-Flag and the other indicated antibodies. The data are representative of three independent experiments (means ± the standard errors of the mean [SEM]). *P ≤ 0.05; **P ≤ 0.01; ***P ≤ 0.001.

It has been demonstrated that the cGAS-STING axis plays a critical role in the induction of type I IFNs in response to ASFV infection (26). To identify ASFV proteins that target cGAS-STING-mediated signaling, we constructed a series of expression clones each encoding an individual ASFV protein. We performed systematic screens for ASFV proteins that could inhibit cGAS-STING mediated activation of the IFNβ promoter and interferon-stimulated response element (ISRE) by reporter assays in HEK293T cells. These efforts led to the identification of 23 candidate ASFV proteins that could antagonize cGAS-STING mediated signaling (data not shown). Among these candidates, ASFV protein QP383R exhibited a strong ability to inhibit cGAS-STING mediated activation of the IFNβ promoter. HEK293T cells were transfected with porcine IFNβ-Luc expression plasmid and pRL-TK plasmid along with FLAG vector or FLAG-tagged-QP383R (FLAG-QP383R), FLAG-cGAS and FLAG-STING expression plasmids. At 24 h post transfection (hpt), the IFNβ promoter activities were determined by using a Dual-Luciferase assay kit. Overexpression of QP383R inhibited cGAS-STING mediated activation of the IFNβ promoter in HEK293T cells (about 25% decreases). In addition, QP383R also inhibited the activation of interferon-stimulated response elements (ISRE) promoter with a more than 32% decrease (**Figure 1A**). Since IRF3 and nuclear factor κB (NF-κB) collaborate to induce the transcription of the *IFNB* gene, we further measured the effects of QP383R on the activation of IRF3 and NF-κB. Consistently, QP383R suppressed cGAS-STING mediated activation of IRF3 and NF-κB (ca. 35 and 25% decreases, respectively) (**Figure 1B**). To investigate whether QP383R affects the expression of IFNβ and IFN-stimulated genes (ISGs), we measured the mRNA expression of antiviral genes in cells that were cotransfected with FLAG-cGAS and FLAG-STING expression plasmids. RT-qPCR experiments indicated that overexpression of QP383R inhibited cGAS-STING-induced transcription of antiviral genes including *IFNB1*, *ISG54*, *ISG15*, and *CXCL10* (**Figure 1C**). Since phosphorylation of TBK1, IRF3, and IκBα are hallmarks of cGAS-STING mediated signaling, we further examined the effects of QP383R on these events. Consistently, overexpression of QP383R dramatically inhibited phosphorylation of TBK1, IRF3, and IκBα in response to cGAS-STING (**Figure 1D**). These data suggest that QP383R is an inhibitor of cGAS-STING mediated signaling.

### 2.2 QP383R inhibits dsDNA-triggered induction of downstream antiviral genes

**Figure 2:**
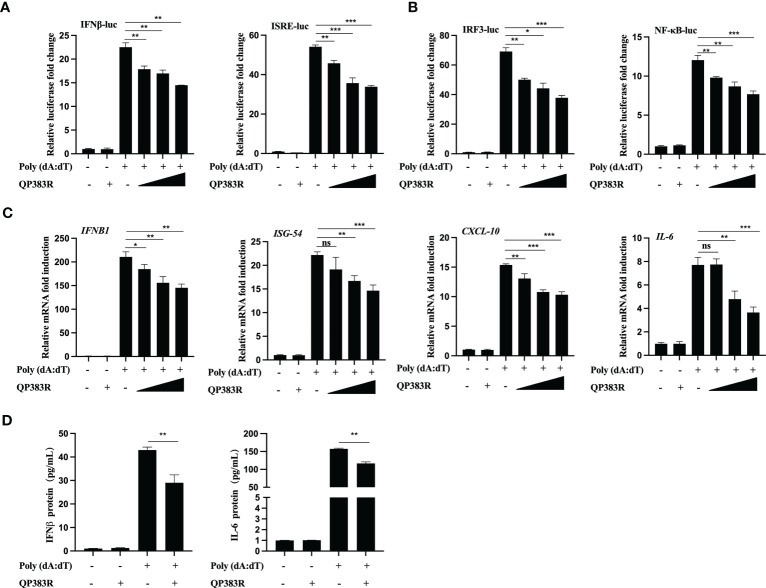
QP383R negatively regulates dsDNA-induced antiviral response. **(A, B)** 3D4/21 cells were transfected with the indicated amounts of QP383R plasmids (0.25, 0.5, and 1.0 μg/ml), and the indicated reporters (50 ng/ml) and pRL-TK (8 ng/ml). Twenty-four hours later, the cells were treated with or without poly(dA:dT) for 12 h, and then the activation of the IFNβ and ISRE promoters **(A)** and IRF3 and NF-κB promoters **(B)** were examined by luciferase assays. **(C)** 3D4/21 cells were transfected with different doses of QP383R expression vectors (0.25, 0.5, and 1.0 μg/ml). At 24 h post-transfection, cells were treated with or without poly(dA:dT) for 12 h, and then *IFNB1*, *ISG54*, *CXCL-10* and *IL-6* mRNAs were detected by q-PCR. **(D)** 3D4/21 cells were transfected with QP383R expression vector or an empty vector for 24 h, and supernatants were harvested after stimulated with or without poly(dA:dT) for 12 h to measure IFNβ and IL-6 productions by ELISA. The data are representative of three independent experiments (means ± the standard errors of the mean [SEM]). *P ≤ 0.05; **P ≤ 0.01; ***P ≤ 0.001.

It has been previously reported that porcine macrophages and monocytes are the primary target cells of ASFV (27–29). To further determine the effect of QP383R on IFNβ promoter activation, the primary porcine alveolar macrophage (PAM) cell line 3D4/21 (CRL-2843) cells were cotransfected with FLAG vector or FLAG-QP383R and porcine IFNβ-Luc expression plasmids, as well as pRL-TK plasmid. At 24 hpt, the cells were treated with the synthetic double-stranded DNA (dsDNA)-mimetic poly(dA:dT) for 12 h, and then the activation of the IFNβ promoter was evaluated. The results showed that QP383R inhibited poly(dA:dT)-induced IFNβ promoter activation in a dose-dependent manner (**Figure 2A**). Overexpression of QP383R also inhibited the activation of the ISRE promoter in a dose-dependent manner in response to transfected poly(dA:dT) (**Figure 2A**). Consistently, QP383R suppressed poly(dA:dT)-mediated activation of IRF3 and NF-kB in a dose-dependent manner (**Figure 2B**). The mRNA expression of antiviral genes in 3D4/21 cells treated with poly (dA:dT) were measured. The results showed that the mRNA levels of *IFNB1*, *ISG54*, *CXCL10* and *IL-6* genes induced upon transfection of poly(dA:dT) were impaired with QP383R overexpression in a dose-dependent manner (**Figure 2C**).

Next, we wanted to verify whether the production of biologically active IFNβ protein and IL-6 protein is decreased by QP383R in poly(dA:dT)-transfected 3D4/21 cells. For this purpose, supernatants from 3D4/21 cells stimulated with poly(dA:dT) were harvested and assessed by enzyme-linked immunosorbent assay (ELISA) for IFNβ and IL-6 production. Consistent with the results obtained with RT-qPCR, when cells were transfected with QP383R and stimulated with poly(dA:dT), the productions of IFNβ and IL-6 proteins were inhibited as compared to empty vector-transduced control cells (ca. 30 and 28% decreases, respectively), confirming that the transduction pathway leading to IFNβ production is impaired in the presence of QP383R (**Figure 2D**). Taken together, these data indicate that QP383R suppresses the activation of the cGAS/STING pathway stimulated by poly(dA:dT) and blocks type I IFN production in porcine cells.

### 2.3 QP383R acts at the level of cGAS

**Figure 3:**
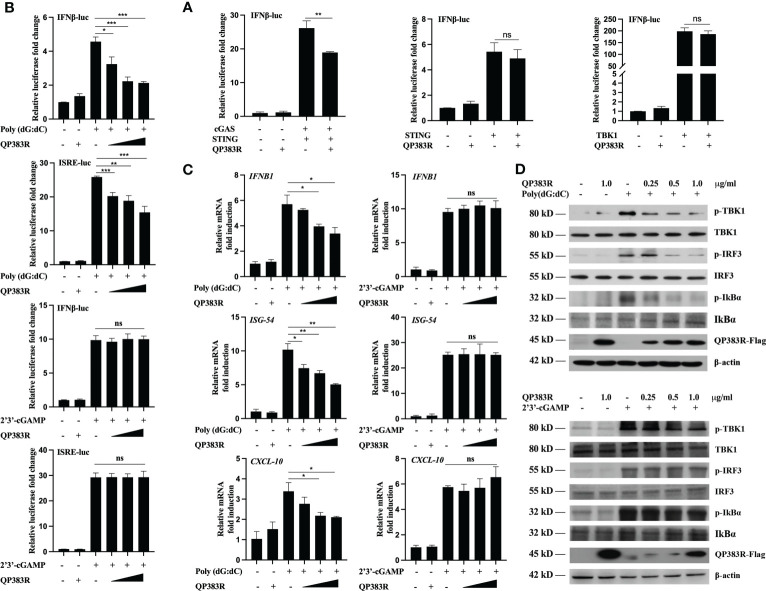
QP383R targets cGAS for antagonizing innate antiviral response. **(A)** HEK239T cells were co-transfected with IFN-luc reporter promoter plasmid, pRL-TK, the expression plasmids for cGAS + STING, STING or TBK1 along with QP383R or empty control plasmid. At 24 h post-transfection, cells were analyzed using dual-luciferase reporter assays. **(B)** 3D4/21 cells were co-transfected with different amounts of QP383R plasmids (0.25, 0.5, and 1.0 μg/ml), pRL-TK, and IFN-luc or ISRE-luc reporter plasmid. Twenty-four hours later, the cells were treated with poly(dG:dC) or 2’3’-cGAMP for 12 h, and the activation of the IFNβ or ISRE promoter was examined by luciferase assays. **(C)** 3D4/21 cells were transfected with different doses of QP383R expression vectors (0.25, 0.5, and 1.0 μg/ml). At 24 h post-transfection, cells were treated with poly(dG:dC) or 2’3’-cGAMP for 12 h, and *IFNB1*, *ISG54*, *CXCL-10* mRNA were detected by q-PCR. **(D)** 3D4/21 cells were transfected with the indicated amounts of QP383R-Flag plasmids for 24 h. Cells were then treated with poly(dG:dC) or 2’3’-cGAMP for 12 h before harvest and analyzed by Western blotting. The data are representative of three independent experiments (means ± the standard errors of the mean [SEM]). *P ≤ 0.05; **P ≤ 0.01; ***P ≤ 0.001.

The observed inhibition of type I IFN production by the ASFV QP383R protein raises the possibility that QP383R targets one or several components of the cGAS-STING signaling pathway. To identify the potential target regulated by QP383R, the porcine IFNβ-Luc and pRL-TK plasmids were co-transfected with FLAG-QP383R expression plasmid and plasmid expressing each component of the cGAS-STING signaling pathway (including cGAS, STING, TBK1, IRF3, IRF3/5D, Ikkα, Ikkβ, and p65) into HEK293T cells. The activation of the IFNβ promoter was determined at 24 hpt. Luciferase reporter assays indicated that overexpression of these component molecules activated IFNβ promoter activity, while overexpression of QP383R protein specially inhibited the activation of the IFNβ promoter induced by cGAS-STING but not STING or TBK1 or other molecules (**Figure 3A**; **Supplementary Figures 1A–E**). These results suggest that QP383R seems to target steps upstream of STING in the cGAS-cGAMP-STING signal pathway.

To further confirm the specific target of QP383R, we measured the activation of the IFNβ promoter and ISRE in 3D4/21 cells stimulated with poly(dG:dC) (another mimic of double-stranded DNA) and 2’3’-cGAMP (an activator of STING downstream of cGAS). Luciferase reporter assays indicated that overexpression of QP383R inhibited poly(dG:dC)- but not 2’3’-cGAMP-induced activation of the IFNβ promoter and ISRE in a dose-dependent manner (**Figure 3B**). In addition, transcription of genes including *IFNB1*, *ISG54* and *CXCL10* following transfection of poly(dG:dC) but not 2’3’-cGAMP, was impaired by QP383R as compared to empty vector-transduced control cells (**Figure 3C**). These results suggest that QP383R seems to regulate the cGAS/STING pathway upstream of cGAMP production. And this conclusion was further confirmed since ectopic expression of QP383R dramatically inhibited the phosphorylation of TBK1, IRF3, and IκBα in response to poly(dG:dC). In contrast, QP383R did not have marked effects on the phosphorylation of TBK1, IRF3, or IκBα induced by 2’3’-cGAMP in 3D4/21 cells (**Figure 3D**). Thus, these findings imply that QP383R targets cGAS for antagonizing innate antiviral response.

### 2.4 QP383R interacts with cGAS

**Figure 4:**
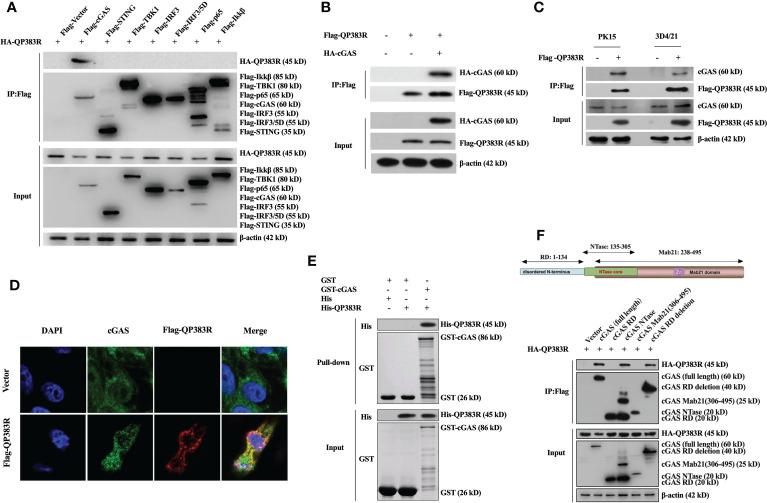
QP383R interacts with cGAS. **(A)** HEK293T cells were transfected with HA-tagged QP383R and Flag-tagged cGAS, STING, TBK1, IRF3, IRF3/5D, p65, Ikkβ or an empty vector for 24 h. Coimmunoprecipitations and immunoblots were performed with the indicated antibodies. **(B)** HEK293T cells were co-transfected with HA-cGAS and Flag-QP383R or their empty vectors for 24 h. Coimmunoprecipitations and immunoblots were performed with the indicated antibodies. **(C)** 3D4/21 and PK15 cells were transfected with Flag-QP383R or an empty vector for 24 h. Cells were then stimulated with poly(dA:dT) for 12 h before harvest and endogenous coimmunoprecipitation and immunoblotting analysis with the indicated antibodies. **(D)** 3D4/21 cells were transfected with Flag-QP383R expression vector or an empty vector (1.0 μg/ml) for 24 h. The colocalization of cGAS and QP383R was observed with confocal microscope (cGAS: green; Flag-QP383R: red; nucleus: blue). **(E)** Purified protein GST-cGAS was incubated with glutathione agarose beads and purified His-QP383R before pull-down assays analysis with the indicated antibodies. **(F)** HEK293T cells were co-transfected with HA-QP383R expression plasmid and Flag-cGAS or its truncation mutants for 24 h before coimmunoprecipitation and immunoblotting analysis with the indicated antibodies.

Given that cGAS is the potential cellular target of QP383R, we next investigated whether QP383R directly interacted with cGAS under physiological conditions. We conducted coimmunoprecipitation (co-IP) experiments to examine whether QP383R is associated with signaling components in cGAS-STING pathways. HEK293T cells were transfected with HA-QP383R expression plasmid and plasmids expressing each of the components in cGAS-STING signaling pathway (including cGAS, STING, TBK1, IRF3, IRF3/5D, p65, and Ikkβ) for 24 h before coimmunoprecipitation and immunoblotting analysis with the indicated antibodies. The results indicated that QP383R was specifically associated with cGAS but not STING, TBK1, IRF3, IRF3/5D (an active mutant of IRF3), p65 or Ikkβ in overexpression system (**Figure 4A**). Consistently, a reverse immunoprecipitation experiment was also performed, and the results showed that cGAS reciprocally coimmunoprecipitated with QP383R in transfected HEK293T cells (**Figure 4B**). Endogenous coimmunoprecipitation experiments further confirmed the association between QP383R and cGAS in PK15 and 3D4/21 cells following poly(dA:dT) transfection (**Figure 4C**). In line with this result, through immunofluorescence assays, we found that QP383R colocalized with cGAS in 3D4/21 cells (**Figure 4D**). Moreover, an *in vitro* glutathione S-transferase (GST) pull-down assay further verified their direct association, indicating a direct interaction between cGAS and QP383R (**Figure 4E**).

Porcine cGAS contains three domains: an RD domain (amino acid residues 1 to 134), a nucleotidyltransferase (NTase) domain (amino acid residues 135 to 305), and a Mab21 domain (amino acid residues 238 to 495) (30, 31). To further study which domain of cGAS is involved in their interaction, we constructed a series of truncation mutants of cGAS. HEK293T cells were cotransfected with HA-QP383R expression plasmid and the indicated truncation mutants of cGAS for 24 h before coimmunoprecipitation and immunoblotting analysis with the indicated antibodies. Coimmunoprecipitation experiments showed that both enzymatically active core (aa135-305) and the deletion of RD domain (aa135-495) of cGAS could interact with QP383R. However, RD domain (aa1-134) or Mab21 domain (aa306-495) of cGAS could not interact with QP383R (**Figure 4F**). Collectively, these results show the specific interaction between cGAS and QP383R, and the enzymatically active core of cGAS is essential for its binding to QP383R.

### 2.5 QP383R impairs DNA binding, dimerization, and enzymatic activity of cGAS through palmitoylation

**Figure 5:**
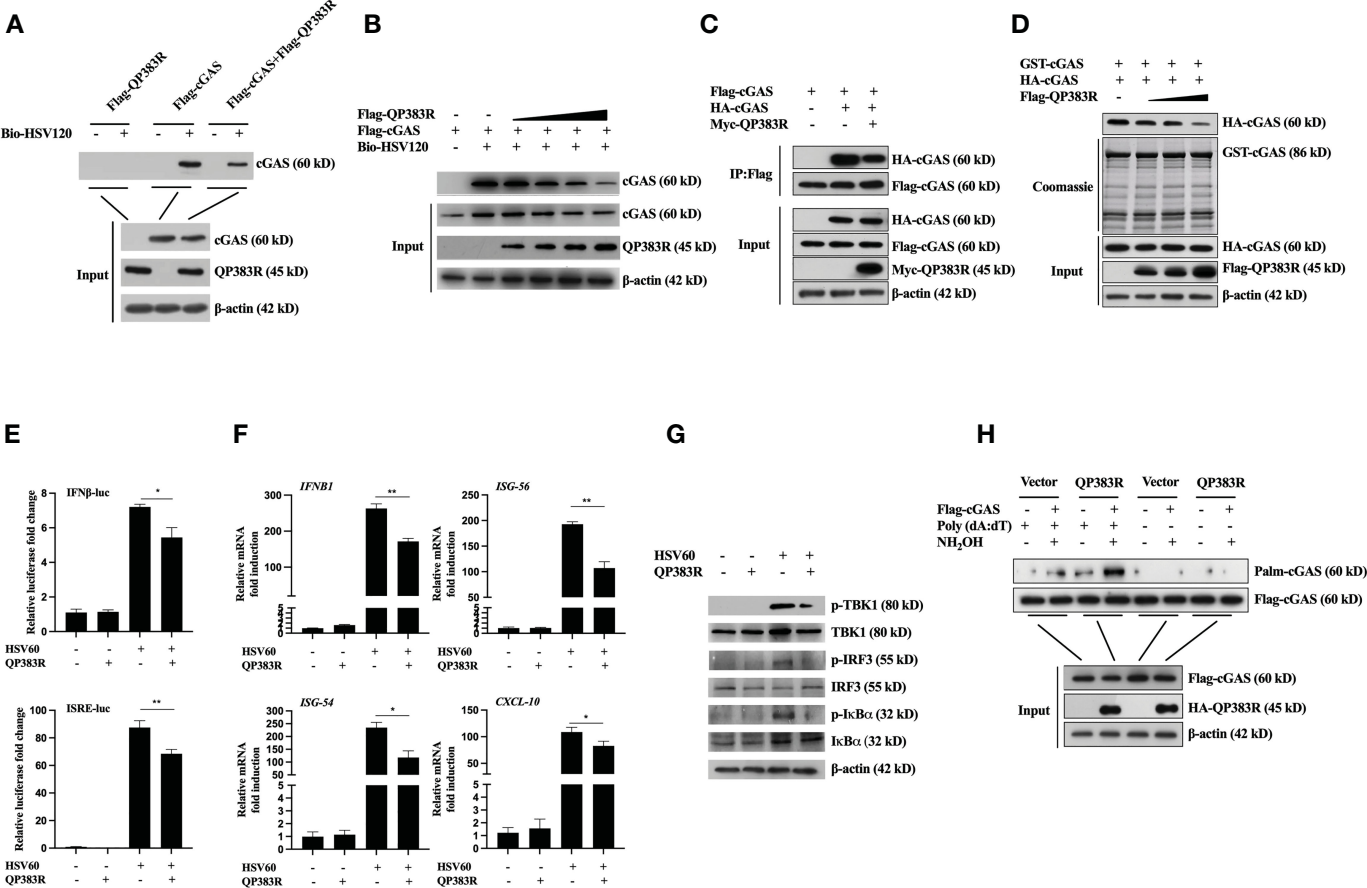
QP383R inhibit cGAS activation. **(A, B)** HEK293T cells were transfected with Flag-QP383R, Flag-cGAS and PRK5-Flag plasmids (1.0 μg/ml). Twenty-four hours later, the cell lysates were incubated with anti-FLAG M2 magnetic beads at 4°C for 4 hours, then eluted with 3 × Flag peptide to purify proteins. The purified proteins were incubated with biotinylated HSV120 and streptavidin-Sepharose beads for *in vitro* pull-down assays. The bound proteins were then analyzed by immunoblots with the indicated antibodies. **(C)** HEK293T cells were transfected with the indicated plasmids for 24 h before co-immunoprecipitation and immunoblotting analysis with the indicated antibodies. **(D)** HEK293T cells were transfected with HA-cGAS (1.0 μg/ml) and Flag-QP383R (0.5, 1.0, and 2.0 μg/ml) for 24 h, cell extracts were incubated with purified GST-cGAS and glutathione agarose beads for 3 hours at 4°C before coomassie staining and/or immunoblot analysis. **(E–G)** Effects of QP383R on cGAMP synthesis induced by transfected HSV60. 3D4/21 cells were transfected with QP383R expression vector or an empty vector (1.0 μg/ml). Twenty-four hours later, cells were stimulated with HSV60 (3.0 μg/ml) for 6 h, and then cell extracts containing cGAMP were delivered to digitonin-permeabilized 3D4/21 cells for 4 h before luciferase assays **(E)**, qPCR analysis **(F)** or western blot analysis **(G)**. **(H)** HEK293T cells were co-transfected with Flag-cGAS (0.5 μg/ml) and HA-QP383R (1.0 μg/ml). At 24 h post-transfection, the cells were harvested after stimulated with or without poly(dA:dT) for 12 h to determine cGAS palmitoylation by IP-ABE assay. The data are representative of three independent experiments (means ± the standard errors of the mean [SEM]). *P ≤ 0.05; **P ≤ 0.01.

In previous reports, the formation of a 2:2 complex with DNA is shown to be important for cGAS activation (32). Therefore, we next determined whether QP383R affected cGAS binding to dsDNA. Purified proteins Flag-QP383R, Flag-cGAS, and PRK5-Flag were incubated with or without biotinylated HSV120 (Bio-HSV120) for *in vitro* pull-down assays. As shown in **Figure 5A**, QP383R did not bind to Bio-HSV120 dsDNA in DNA-pull-down assays. However, QP383R dramatically inhibited the binding of cGAS to Bio-HSV120 dsDNA (**Figure 5A**). The inhibitory effect of QP383R on cGAS binding to dsDNA was in a dose-dependent manner (**Figure 5B**). These results suggest that QP383R impairs cGAS binding to dsDNA.

Previously, it has been shown that cGAS self-association and oligomerization are important for its activation after binding to dsDNA (33, 34). Since QP383R inhibits cGAS binding to dsDNA, we speculated that QP383R also affected cGAS dimerization. To test this hypothesis, we conducted coimmunoprecipitation experiments to examine whether QP383R inhibits self-association of cGAS. Co-IP experiments indicated that Flag-cGAS interacted with HA-cGAS, while this self-association was inhibited with the overexpression of QP383R (**Figure 5C**). Consistently, QP383R markedly inhibited cGAS dimerization in a dose-dependent manner in Co-IP assays (**Figure 5D**). The results reveal that cGAS dimerization is inhibited by QP383R.

As previously described (35, 36), the extracts from DNA-transfected cells contain cGAMPs, which activate the IFNβ and ISRE promoters, trigger the expression of *IFNB1*, *ISG56*, *ISG54* and *CXCL10* genes, and induce TBK1, IRF3, and IκBα phosphorylation. To elucidate the mechanisms on how QP383R antagonizes innate antiviral response, we next assessed whether QP383R affected cGAS enzymatic activity. Using a previously developed bioassay (37), we transfected 3D4/21 cells with or without QP383R for 24 h, then stimulated with HSV60 (another mimic of double-stranded DNA) to stimulate cGAMP production. Cell lysates were digested with DNase and then boiled to remove DNA and proteins, and the supernatant containing cGAMPs were collected by centrifugation and added to 3D4/21 cells with digitonin, followed by measurement of IFNβ expression, which indirectly represents the cGAMP level. As shown in **Figure 5E**, the activation of IFNβ and ISRE promoter in 3D4/21 cells was dramatically increased with the addition of the supernatant, indicating that cGAS was activated to produce a large amount of cGAMPs. However, QP383R overexpression significantly reduced the activation of IFNβ and ISRE promoters, suggesting that QP383R restricted cGAS activity and cGAMP production (**Figure 5E**). Consistently, QP383R overexpression inhibited the cGAMP-mediated expression of antiviral genes, including *IFNB1*, *ISG56*, *ISG54* and *CXCL10* (with decreases of ca. 36, 45, 50, and 26%, respectively). (**Figure 5F**). The same results were obtained when we assessed the phosphorylation level of TBK1, IRF3, and IκBα (**Figure 5G**). TBK1, IRF3, and IκBα were apparently phosphorylated after transfection of the supernatant, whereas QP383R overexpression markedly inhibited TBK1, IRF3, and IκBα phosphorylation, confirming the negative role of QP383R on the enzymatic activity of cGAS. Taken together, our findings suggest that QP383R impairs the synthesis of cGAMPs by inhibiting DNA binding and dimerization of cGAS.

In the cGAS-STING signaling pathway, it has been reported that palmitoylation of cGAS inhibits DNA binding and cGAS dimerization, and also restricts its enzymatic activity (38). Next, we wanted to verify whether QP383R regulates the palmitoylation of cGAS. We detected cGAS palmitoylation using IP-ABE assay. We replaced the palmitoylation modification of cGAS with biotin modification, and analyzed the changes in the palmitoylation levels of cGAS by western blot using the affinity of biotin and streptavidin. As shown in **Figure 5H**, the protein samples treated with NH_2_OH developed a cGAS band, indicating that cGAS was modified by palmitoylation. Interestingly, we found that QP383R promoted elevation of the palmitoylation level of cGAS stimulated with poly(dA:dT) (**Figure 5H**).

These results suggest that QP383R inhibits DNA binding, cGAS dimerization, and the enzymatic activity of cGAS due to palmitoylation of cGAS promoted by QP383R.

### 2.6 Amino acids 284-383 in QP383R are responsible for its inhibitory effect on IFN-I production

**Figure 6:**
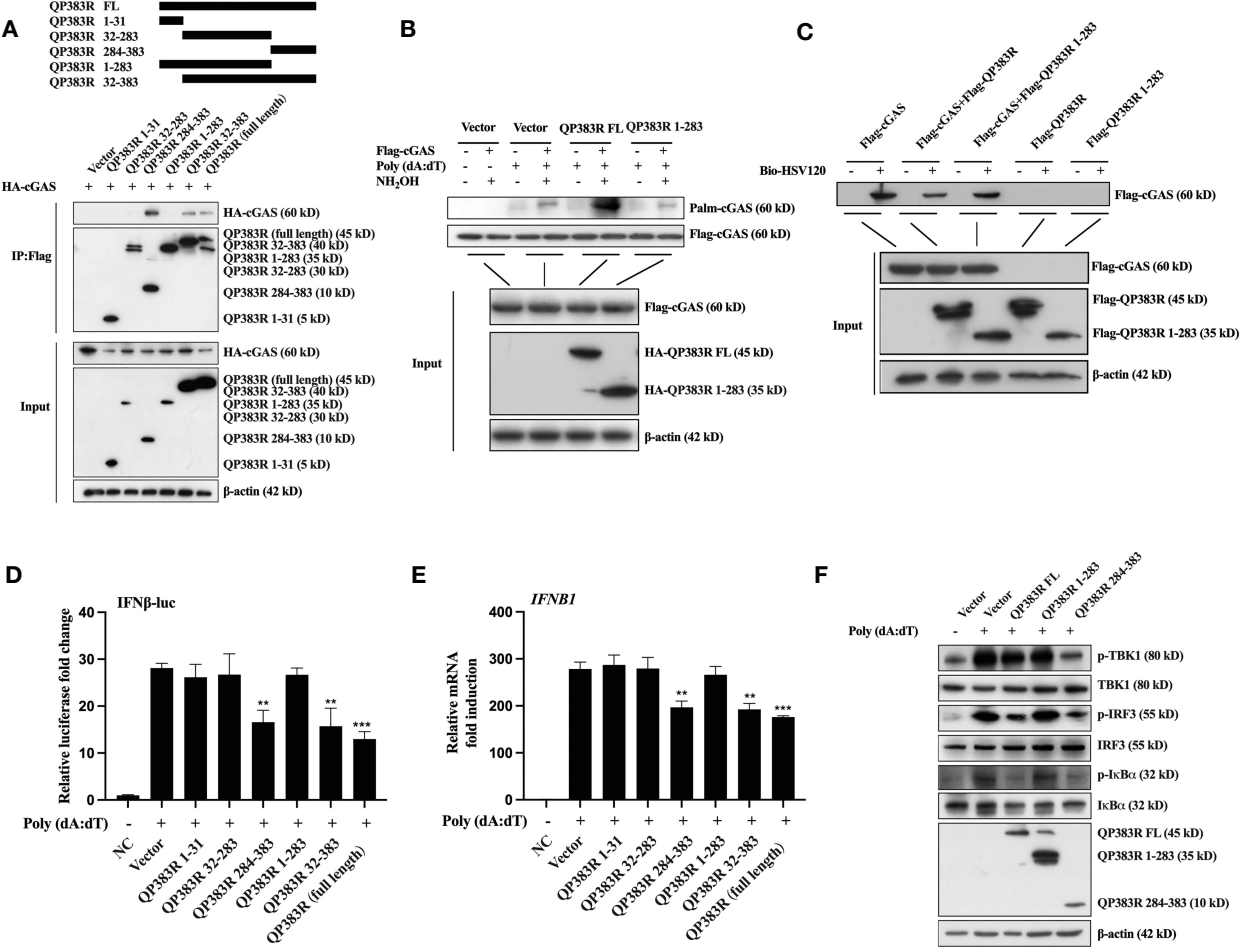
284-383aa of QP383R was essential for its inhibitory effect. **(A)** HEK293T cells were co-transfected with HA-cGAS expression plasmid and Flag-QP383R or its truncation mutants for 24 h before coimmunoprecipitation and immunoblotting analysis with the indicated antibodies. **(B)** HEK293T cells were co-transfected with Flag-cGAS (0.5 μg/ml) and HA-QP383R FL (1.0 μg/ml) or HA-QP383R 1-283 (1.0 μg/ml). At 24 h post-transfection, the cells were harvested after stimulated with or without poly(dA:dT) for 12 h to determine cGAS palmitoylation by IP-ABE assay. **(C)** The purified proteins were incubated with the indicated biotinylated HSV120 and streptavidin-Sepharose beads for *in vitro* pull-down assays. The bound proteins were then analyzed by immunoblots with the indicated antibodies. **(D)** 3D4/21 cells were transfected with the indicated plasmids (1.0 μg/ml), IFN-luc reporter promoter plasmid (50 ng/ml), and pRL-TK (8 ng/ml). Twenty-four hours later, the cells were treated with or without poly(dA:dT) for 12 h, and then the activation of the IFNβ promoter was examined by luciferase assays. **(E)** 3D4/21 cells were transfected with the indicated plasmids (1.0 μg/ml). At 24 h post-transfection, cells were treated with or without poly(dA:dT) for 12 h, and IFNB1 mRNA was detected by q-PCR. **(F)** 3D4/21 cells were transfected with the indicated plasmids (1.0 μg/ml). At 24 h later, the cells were stimulated with or without poly(dA:dT) for 12 h. The cells were then harvested and lysed for Western blot analysis to determine the levels of p-TBK1, p-IRF3 and p-IκBα. The data are representative of three independent experiments (means ± the standard errors of the mean [SEM]). **P ≤ 0.01; ***P ≤ 0.001.

QP383R is a non-structural protein of ASFV, which is known as an uncharacterized protein. QP383R is highly conserved among virulent and nonvirulent isolates and consists of 383 amino acids, which contains a predicted “aminotransferase class-V” motif (aa32-283). To identify the key domains in QP383R that were essential for its interaction with cGAS, a series of truncated mutants were generated, including FLAG-QP383R 1-31aa, FLAG-QP383R 32-283aa, FLAG-QP383R 284-383aa, FLAG-QP383R 1-283aa and FLAG-QP383R 32-383aa. HEK293T cells were cotransfected with FLAG vector, FLAG-QP383R, or each of the FLAG-QP383R mutant expression plasmids, and HA-cGAS expression plasmid. And then, the cell lysates were immunoprecipitated with anti-FLAG antibody and analyzed by Western blotting. The co-IP result showed that only the amino acids 284-383 in QP383R retained the interaction with cGAS, whereas other QP383R mutant expression plasmids without 284-383aa abolished the binding with cGAS (**Figure 6A**), suggesting that the region of amino acids 284-383 in QP383R is essential for the interaction between QP383R and cGAS.

Based on this observation, we speculate that the immunosuppressive function of QP383R maybe need its interaction with cGAS. To test this hypothesis, we cotransfected HEK293T cells with HA vector, HA-QP383R, or HA-QP383R-1-283 (deletion of the amino acid 284 to 383 region of QP383R) mutant expression plasmid and stimulated with poly(dA:dT) for 12 h, and IP-ABE assay was used to analyze the changes in the palmitoylation levels of cGAS. As expected, QP383R promoted the palmitoylation of cGAS, while the deletion of the amino acid 284 to 383 region of QP383R completely abrogated the promotion of cGAS palmitoylation (**Figure 6B**). These results suggest that the amino acid 284 to 383 region in QP383R is essential for QP383R to modulate cGAS palmitoylation, implying that QP383R promotes cGAS palmitoylation needs its amino acid 284-383 region to interact with cGAS first.

To further confirm whether the deletion of the amino acid 284 to 383 region in QP383R loses its inhibitory effect on dsDNA binding to cGAS, HEK293T cells were cotransfected with FLAG-QP383R or FLAG-QP383R-1-283 and HA vector or HA-cGAS. Then cell lysates were incubated with biotinylated HSV120 for 1 hour followed by incubation with streptavidin agarose for 2 h. The agarose beads were analyzed by immunoblotting. As expected, QP383R impaired the interaction between cGAS and HSV-120 (a double-stranded DNA). While, deletion of amino acid 284 to 383 region in QP383R lost its inhibitory activity against dsDNA binding to cGAS (**Figure 6C**).

The amino acids 284 to 383 region of QP383R was further clarified to be crucial for its immunosuppressive function. 3D4/21 cells were cotransfected with FLAG vector or FLAG-QP383R or FLAG-QP383R mutant expression plasmid along with porcine IFNβ-Luc expression plasmid and pRL-TK plasmid. At 24 hpt, the cells were treated with poly(dA:dT) for 12 h, and the activation of the IFNβ promoter was evaluated. QP383R, QP383R 284-383aa and QP383R 32-383aa but not other QP383R mutant expression plasmids suppressed IFNβ promoter activation (**Figure 6D**), suggesting that the C-terminal domain of QP383R (aa284-383) is responsible for blocking cGAS-STING signaling pathway activation.

We also measured the mRNA expression of IFNβ in 3D4/21 cells. 3D4/21 cells were cotransfected with FLAG vector or FLAG-QP383R or FLAG-QP383R mutant expression plasmid and stimulated with poly(dA:dT) for 12 h. Consistently, only QP383R, QP383R 284-383aa, and QP383R 32-383aa reduced the transcription of *IFNB1* (**Figure 6E**). In line with these results, through Western blot, we found that only QP383R and QP383R 284-383aa inhibited the phosphorylation of TBK1, IRF3 and IκBα in response to poly(dA:dT) (**Figure 6F**).

Taken together, these results indicate that QP383R has an activity to suppress the host antiviral response through blocking type I IFN production, while the amino acid 284 to 383 region in the C-terminal domain of QP383R is indispensable for its inhibitory function against type I IFN production.

## 3 Discussion

Type I interferons represent one of the first lines of defense against the invasion of virus. When a virus infects hosts, various pattern recognition receptors recognize pathogen-associated molecular patterns and result in the activation of innate immune signaling pathways to produce IFN-I (39). As an important axis in activating innate immune signaling pathways to produce IFN-I, the cGAS-STING signaling axis could not only detects pathogenic DNA to trigger an innate immune reaction involving a strong type I interferon response against microbial infections, but also can be activated by endogenous DNA, including extranuclear chromatin resulting from genotoxic stress and DNA released from mitochondria (40).

As the cGAS-STING axis plays a crucial role in host antiviral defense (41), many viruses have evolved various mechanisms to antagonize this signaling pathway for efficient infection and replication (42). For example, HCMV tegument protein UL82 contributes to HCMV immune evasion by inhibiting the cellular trafficking and activation of MITA/STING to evade antiviral immunity (35). UL83 inhibits gamma-interferon-inducible protein 16 (IFI16)- and cGAS-mediated DNA sensing for immune evasion (43). PPRV infection impairs the interaction of IRF3 with TBK1 and inhibits IRF3 nuclear translocation, resulting in the suppression of IFN synthesis (44). Virulent poxviruses suppresses host type I IFN production by preventing STING activation (45). Similar to many other DNA viruses, the cGAS-STING axis also plays a crucial role in ASFV-induced host antiviral defense (26, 46). Meanwhile, several proteins encoded by ASFV could antagonize cGAS-STING signaling pathway through different mechanisms for efficient infection and replication (47–49). For example, it has been demonstrated that ASFV protein pA137R negatively regulates the cGAS-STING-mediated IFNβ signaling pathway *via* the autophagy-mediated lysosomal degradation of TBK1 (50). EP364R and C129R of ASFV cleave 2’3’-cGAMP to inhibit the cGAS-STING signaling pathway (51). Moreover, DP96R of ASFV China 2018/1 strain subverts type I IFN production in the cGAS sensing pathway by inhibiting both TBK1 and IKKβ (52). However, whether other ASFV proteins are involved in antagonization of innate antiviral response are largely unclear. Here we identified ASFV protein QP383R as an inhibitor of cGAS-STING-mediated innate antiviral response. Overexpression of QP383R inhibited cGAS-induced activation of the IFNβ promoter and ISRE promoter. Consistently, QP383R inhibited cytosolic dsDNA-induced production of type I IFNs and transcription of downstream antiviral effector genes. These results suggest that ASFV QP383R acts to antagonizing cGAS-STING-mediated innate antiviral immune response, and has the potential to help ASFV achieve immune escape.

As a nonredundant cytosolic DNA sensor, cGAS plays an important role in anti-DNA virus. Therefore, different antagonistic mechanisms targeting cGAS have been identified in various viruses. For example, herpes simplex virus 1 (HSV-1) tegument protein UL37 has been reported to deamidate cGAS, which impairs the ability of cGAS to catalyze cGAMP synthesis (53). HSV-1 protein UL41 has been reported to directly degrade cGAS mRNA to inhibit antiviral signaling (54). ICP27 targets the TBK1-activated MITA/STING signalosome to inhibit antiviral response (55). Kaposi sarcoma herpesvirus (KSHV) protein ORF52 and cytoplasmic isoforms of LANA counteract cGAS-STING pathways through binding to cGAS (56, 57). However, to date, knowledge of the ASFV proteins that regulate cGAS function is limited (58). In this report, several lines of evidence suggest that QP383R directly targets cGAS. Firstly, overexpression of QP383R inhibited cGAS-STING- and dsDNA-, but not cGAMP-induced induction of type I IFNs in 3D4/21 cells and HEK293T cells, suggesting that QP383R targets components upstream of cGAMP. Secondly, co-IP experiments indicated that QP383R was reciprocally associated with cGAS *in vivo* and *in vitro*. Immunofluorescence assays further confirmed the colocalization of QP383R with cGAS in 3D4/21 cells. Thirdly, an *in vitro* GST pull-down assay further verified the direct interaction between cGAS and QP383R.

Extensive studies have revealed the essential roles of cGAS in multiple biological processes, including pathogen invasion and autoimmune diseases. The function of cGAS must be tightly controlled, preventing both over inhibition, which leads to silenced innate immune responses and pathogen invasion, and over activation, which may lead to auto-immune or chronic inflammatory diseases. cGAS activity is reported to be regulated by various PTMs, including ubiquitylation, sumoylation, glutamylation, phosphorylation, acetylation and palmitoylation (59). As a PTM, palmitoylation usually occurs on membrane-associated proteins to regulate their subcellular localization or conformational state. For the first time, cGAS is found to have palmitoylation, but cGAS palmitoylation does not affect its subcellular localization (38). Palmitoylation is a common regulatory mechanism in conformational change. Shi et al. have found that human cGAS palmitoylation alters the interaction between specific amino acid residues and causes conformational changes through MD simulation and biochemical verification. In our study, we found that porcine cGAS also had palmitoylation. Porcine cGAS is not palmitoylated in the resting state, while cGAS palmitoylation appears under stimulation by cytosolic double-stranded DNA. Overexpression of QP383R promoted elevation of the palmitoylation level of cGAS stimulated with poly(dA:dT), which inhibited DNA binding, dimerization, and the enzymatic activity of cGAS. In our study, the palmitoylation modification of porcine cGAS was found for the first time, and cGAS palmitoylation was identified as a novel inhibitory mechanism of the innate immune response to ASFV. Because the structure or function of QP383R is still unknown, whether QP383R has palmitoylase activity, or recruits palmitoyltransferase to interact with cGAS, or inhibits the interaction between depalmitoylase and cGAS to promote cGAS palmitoylation needs further investigation.

Live attenuated vaccines, developed by deleting one or more of their specific virulence-associated and immunosuppressive genes in the genome of the virulent strains, have been shown to elicit protection against experimental challenge with virulent parental viruses (60–66). These findings suggest that the development of attenuated ASFV recombinant viruses through the genetic manipulation of specific gene(s) could be the most promising strategy for vaccine development so far. As an immunosuppressive factor, QP383R might be a potential target for LAVs design. It has been reported that QP383R is an inhibitor of inflammatory response and deletion of the QP383R genes (ASFV-ΔQP383R) from the highly virulent ASFV CN/GS/2018 strain results in partly viral attenuation in pigs (67). In our study, we found that QP383R is an immunosuppressive factor, inhibited innate antiviral response by reducing the production of type I IFNs. Meanwhile, the amino acid 284 to 383 region of QP383R is indispensable for its inhibitory function against type I IFN production. Therefore, we assume that existing LAVs with the deletion of QP383R gene or deletion/mutation of its key domain (amino acid 284 to 383) at the same time, would dramatically induce the production of type I IFNs, which might play an important role in improving vaccine efficacy.

Interestingly, a recent study shows that the ASFV CN/GS/2018 strain lacking the QP509L and QP383R genes (ASFV-ΔQP509L/QP383R) is completely attenuated *in vivo* in pigs. However, the recombinant ASFV-ΔQP509L/QP383R does not induce protection against lethal ASFV challenge due to its lower levels of type I interferon induction in porcine macrophages (67), which seems to be in contrast to our findings that QP383R inhibits type I interferon production. We speculate that the lower levels of type I interferon induction in porcine macrophages infected with ASFV-ΔQP509L/QP383R is due to its low- or no-replication phenotype. On the other hand, it is reported that the same genes might have different functions in different ASFV strains (8, 61, 63, 64, 66). Therefore, QP383R could play a different function in ASFV CN/GS/2018 strain and ASFV Pig/HLJ/2018 strain.

**Figure 7:**
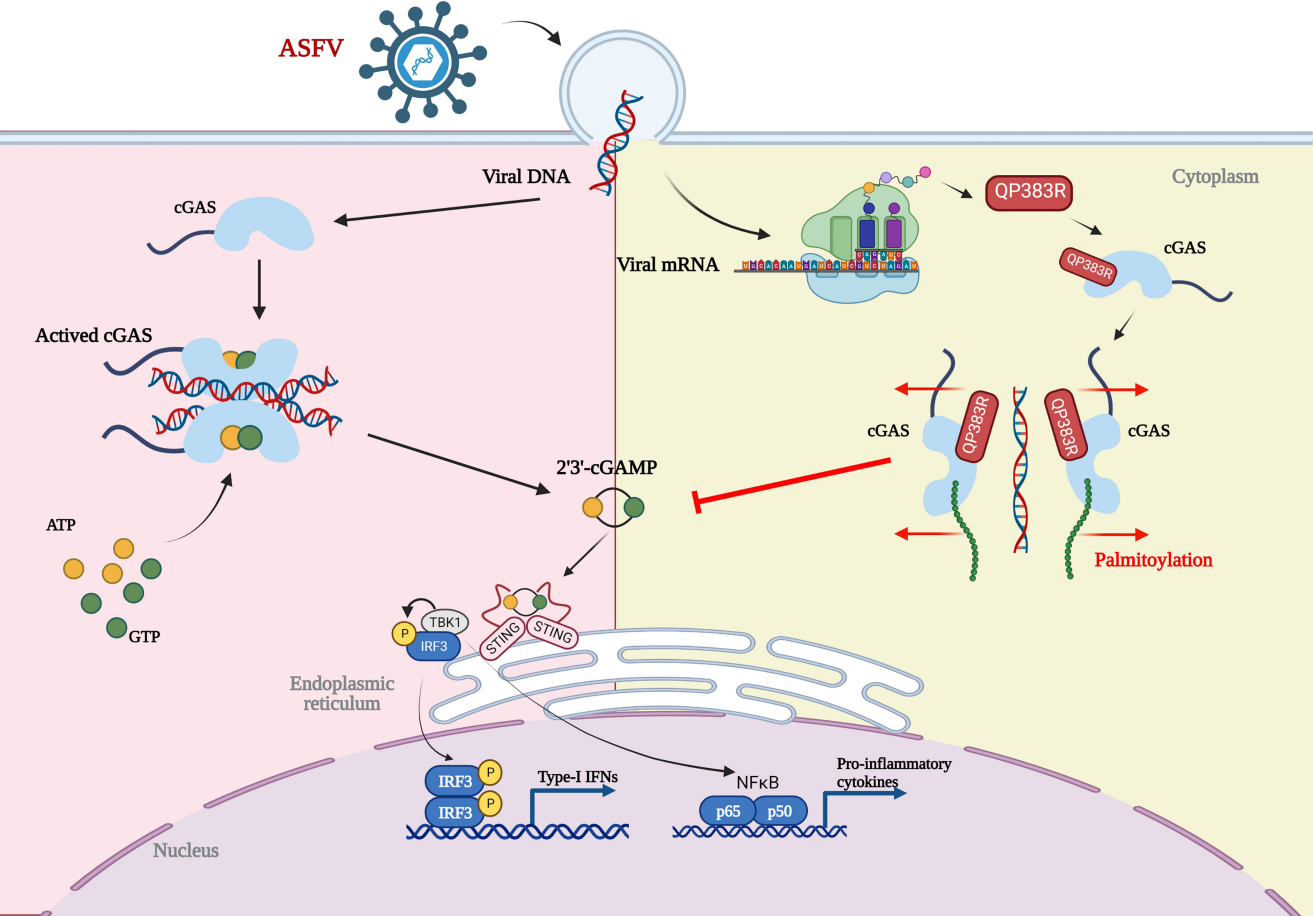
Model of the molecular mechanism for QP383R to inhibit IFN-I production. Upon ASFV infection, QP383R is expressed. QP383R interacts with cGAS to inhibit cGAS dimerization, DNA binding, and enzymatic activity *via* promoting its palmitoylation, resulting in the suppression of IFN-I production. Figure was created with BioRender (https://biorender.com).

Based on our results, we propose a working model on QP383R-mediated immune evasion of ASFV (**Figure 7**). Upon ASFV infection, QP383R is expressed and recruited to cGAS. Subsequently, QP383R uses its amino acid 284-383 to interact with the enzymatically active core of cGAS (aa135-305), and promotes the palmitoylation of cGAS. While, cGAS palmitoylation alters the interactions between specific amino acid residues and causes a conformational change, leading to the inhibition of cGAS DNA binding and dimerization, and the synthesis of cGAMPs. This causes the inhibition of type I IFNs production and innate antiviral response. In summary, these findings expand our knowledge on regulatory mechanisms of the cGAS-STING signal pathway, as well as the strategies of immune evasion by ASFV, which may facilitate the development of the vaccines and therapeutics against ASFV infection.

## 4 Materials and methods

### 4.1 Cells

Human embryonic kidney 293T (HEK293T) cells (ATCC CRL-3216™) and porcine kidney 15 (PK-15) cells (ATCC CCL-33) were cultured in Dulbecco’s modified Eagle medium (DMEM; Gibco). A porcine alveolar macrophage cell line 3D4/21 cells (ATCC CRL-2843, which is established by transformation of PAMs with SV40 large T antigen) were maintained in RPMI medium 1640 (Gibco). Cells were supplemented with 10% heat-inactivated fetal bovine serum (FBS; Gibco) and 1% antibiotic/antimycotic (Gibco) and incubated in a humid 5% CO_2_ incubator at 37°C.

### 4.2 Reagents and antibodies

Poly(dA:dT) naked, Poly(dG:dC) naked, 2’3’-cGAMP, and HSV-60 naked were acquired from Invivogen. Lipofectamine 3000 transfection kit (Invitrogen); jetPRIME transfection reagent (Polyplus-transfection); dual-luciferase reporter assay system (Promega); digitonin (Sigma); streptavidin agarose (Solarbio); hydroxylamine solution (HAM, Sigma-Aldrich); EZ-Link BMCC-Biotin (ThermoFisher). The commercial antibodies used in this study included rabbit FLAG mAb (#14793), mouse FLAG mAb (#8146), mouse His mAb (#2366), rabbit TBK1/NAK mAb (#3504), rabbit phosphorylated-TBK1/NAK (p-TBK1) mAb (#3504), mouse IRF3 mAb (#10949), rabbit IRF3 mAb (#4302), rabbit p-IRF3 (Ser 396) mAb (#4947), mouse IκBα mAb (#4814), and rabbit p-IκBα (Ser 32) mAb (#2859). Streptavidin-HRP antibody (#3999) were acquired from Cell Signaling Technology (Boston, MA, USA). The rabbit cGAS pAb (HA500023) was obtained from HuaAn Biotechnology (Hangzhou, Zhejiang, China). The rabbit p-IRF3 (Ser 396) mAb (SAB4504031), rabbit HA mAb (H6908), and mouse β-actin mAb (A1978) were purchased from Sigma-Aldrich (St. Louis, MO, USA).

### 4.3 RNA extraction and real-time PCR

Total RNAs were extracted from treated cells with TRIzol reagent (CWBIO, China), and 1μg of total RNAs were then reverse transcribed to cDNA using HiFiScript cDNA Synthesis Kit (CWBIO, China) according to the manufacturer’s instructions. Real-time PCR analysis was performed by using M5 HiPer Real-time PCR Super Mix (Mei5Bio, China) in a ViiA 7 real-time PCR system (Applied Biosystems). The gene-specific primers for human *IFNB1*, *ISG15*, *ISG54*, and *CXCL10*, and porcine *IFNB1*, *ISG54*, *ISG56*, *IL-6* and *CXCL10* were listed in **Supplementary Table 1**. The level of gene mRNA was normalized according to the amount of endogenous control glyceraldehyde-3-phosphate dehydrogenase (GAPDH) expression.

### 4.4 ELISA

The IFNβ and IL-6 protein levels in cell culture supernatants were measured using pig IFNβ ELISA kits (CUSABIO, China) and pig IL-6 ELISA kits (CUSABIO, China) respectively in accordance with the manufacturer’s instructions.

### 4.5 Plasmid construction

The QP383R gene of ASFV Pig/HLJ/2018 (GenBank submission No. MK333180) was synthesized by BGI, and cloned into the pcDNA3.1(+), PRK5-HA, PRK5-Flag, pCMV-Myc or pET-32a vectors by standard molecular biology techniques. ASFV QP383R’s truncation mutants including QP383R 1-31aa, QP383R 32-283aa, QP383R 284-383aa, QP383R 1-283aa, and QP383R 32-383aa were cloned into PRK5-Flag, respectively, using seamless assembly cloning kit (cloneamarter, USA). Porcine cGAS, STING, TBK1, IRF3, IRF3/5D, p65, Ikkβ expression plasmids and pGL3-IFNβ-luc, pGL3-ISRE-luc, pGL3-IRF3-luc, pGL3-NFκB-luc reporter vectors were constructed and preserved in our laboratory previously (68, 69). Truncation mutants of porcine cGAS in this article including cGAS RD (1-134aa), cGAS NTase (135-305aa), cGAS Mab21 (306-495aa), and cGAS RD deletion (135–496) were amplified using the cGAS plasmid as a template and cloned into PRK5-Flag respectively, using seamless assembly cloning kit (cloneamarter, USA). The primers for amplification of plasmids were listed in **Supplementary Table 2**.

### 4.6 Luciferase reporter assays

HEK293T cells and 3D4/21 cells seeded in 24-well plates were transfected with the constructed plasmids using Lipofectamine 3000 according to the manufacturer’s protocol, and pRL-TK reporter plasmid was transfected as an internal control. After 24 h, the cells were treated with or without the synthetic double-stranded DNA-mimetic for 12 h. Cell extracts were prepared and analyzed for firefly and Renilla luciferase activities using a dual-luciferase reporter assay kit (Promega) according to the manufacturer’s instructions.

### 4.7 Western blot

HEK293 cells, or 3D4/21 cells were lysed in radioimmunoprecipitation assay (RIPA) lysis buffer (CWBIO) with 100 U of proteinase inhibitors (CWBIO) and 20 μM NaF on ice for 20 min. Protein levels were quantified using bicinchoninic acid assay. Similar amounts of protein from each extract were separated by SDS-12% PAGE and transferred to polyvinylidene difluoride membranes (Millipore). Membranes were blocked with 5% skim milk in PBS with 0.05% Tween 20, followed by incubation overnight at 4°C with the indicated antibodies. The membranes washed three times with PBST, and then incubated with the appropriate secondary antibody for 1 h at room temperature. Signals were visualized using enhanced chemiluminescence.

### 4.8 Coimmunoprecipitation

HEK293 cells, or 3D4/21 cells were lysed in l ml NP-40 lysis buffer (50 mM Tris-HCl pH 7.5, 150 mM NaCl, 5 mM EDTA, 1% Nonidet P-40, 10% glycerin) with 100 U of proteinase inhibitors and 20 M NaF for 20 min on ice. Cell lysates were clarified by centrifugation at 12,000 rpm for 10 minutes (4°C). For each immunoprecipitation, the cell lysates was incubated with anti-FLAG M2 magnetic beads at 4°C for 4 hours or overnight. The protein-bound beads were then collected and washed three times with lysis buffer.

### 4.9 Fluorescent confocal microscopy

3D4/21 cells were transfected with Flag-QP383R or PRK5-Flag for 24 h and fixed in 4% paraformaldehyde for 10-20 min at room temperature. After being washed three times with PBS, the cells were permeabilized in 0.1% Triton X-100 for 5 min on ice. After being washed three times with PBS, the cells were incubated with 1% BSA for 1 h at room temperature. The cells were incubated with rabbit anti-cGAS MAb (1:500) and mouse anti-Flag MAb (1:1000) overnight at 4°C. Following incubation with Alexa Fluor 555-conjugated goat anti-mouse IgG antibody (1:500) or Alexa Fluor 488-conjugated goat anti-rabbit IgG antibody (1:500) for 1 h at room temperature in the dark, the cells were washed with PBS. Subsequently, the cells’ nuclei were stained with 4’,6-diamidino-2-phenylindole (DAPI) (1:1,000) and observed by laser confocal microscopy.

### 4.10 DNA oligonucleotides

HSV120: 5’-AGACGGTATATTTTTGCGTTATCACTGTCCCGGATTGGACACGGTCTTGTGGGATAGGCATGCCCAGAAGGCATATTGGGTTAACCCCTTTTTATTTGTGGCG GGTTTTTTGGAGGACTT-3’.

### 4.11 DNA pull-down assays

Bio-HSV120 upstream and downstream primers were synthesized by Sangon. HEK293T cells transfected with the indicated plasmids were lysed with lysis buffer (20 mM Tris-HCl pH 7.4, 150 mM NaCl, 5 mM EDTA, 1% Nonidet P-40, 10% glycerin) and cell lysates were incubated with anti-FLAG M2 magnetic beads at 4°C for 4 hours. The protein-bound beads were then collected and eluted with 3×Flag peptide to purify proteins. The purified proteins were incubated with biotinylated HSV120 for 2 hours at 4°C, followed by incubation with streptavidin-Sepharose beads for 3 hours at 4°C. The agarose beads were collected and washed three times with lysis buffer before immunoblotting analysis with the indicated antibodies.

### 4.12 GST pull-down assay

Purified GST-cGAS were incubated with glutathione agarose beads at 4°C for 1 hour, followed by incubation with purified His-QP383R for 3 hours at 4°C. The beads were washed three times each with lysis buffer (50 mM Tris-HCl pH 7.5, 150 mM NaCl, 5 mM EDTA, 1% Nonidet P-40, 10% glycerin, 100 U proteinase inhibitors, 20 M NaF), and then mixed with an equal volume of 2 × SDS loading buffer and boiled for 10 min. The input/elutes were resolved by SDS-PAGE and analyzed by coomassie staining and/or immunoblot analysis.

### 4.13 cGAMP activity assays

3D4/21 cells were transfected with FLAG vector or FLAG-QP383R for 24 h, then treated with HSV60 (3 μg/ml) for 6 hours. Cell extracts were then prepared and heated at 95°C for 5 min to denature most proteins, which were removed by centrifugation. The supernatants containing cGAMP were delivered to 3D4/21 cells pretreated with digitonin permeabilization solution (50 mM HEPES pH 7.0, 100 mM KCl, 3 mM MgCl_2_, 0.1 mM DTT, 85 mM Sucrose, 0.2% BSA, 1 mM ATP, 0.1 mM GTP and 10μg/ml digitonin) at 37°C for 30 min. Four hours later, the cells were collected for Luciferase, qPCR analysis, or western blot.

### 4.14 IP-ABE assays

The *in vitro* palmitoylation assay was performed as previously described, with minor modifications (70, 71). HEK293T cells transfected with the indicated plasmids were lysed with ABE lysis buffer (50 mM Tris-HCl pH 7.5, 150 mM NaCl, 1% Nonidet P-40, 10% glycerin, 1 mM PMSF, 50 mM NEM, 100 U proteinase inhibitors) for 20 min on ice. The cell lysate was incubated with anti-FLAG M2 magnetic beads at 4°C overnight in dark. The protein-bound beads were then collected and washed three times with ABE lysis buffer (pH 7.2), and were then divided into two equal groups: group added with HAM (+HAM) as experimental group, and group added without HAM (-HAM) as control group. The samples were incubated separately with HAM buffer (50 mM Tris-HCl pH 7.2, 150 mM NaCl, 1% Nonidet P-40, 10% glycerin, 1 mM PMSF, 50 mM NEM, 100 U proteinase inhibitors, 1 M 50% HAM) and ABE lysis buffer (pH 7.2) for 50 min at room temperature. The protein-bound beads were washed with ABE lysis buffer (pH 6.2), following incubation with Biotin-BMCC buffer (50 mM Tris-HCl pH 6.2, 150 mM NaCl, 1% Nonidet P-40, 10% glycerin, 1 mM PMSF, 50 mM NEM, 100 U proteinase inhibitors, 1 μM Biotin-BMCC) for 45 min at 4 °C. After washed by ABE lysis buffer (pH 7.5) for three times, the protein-bound beads were mixed with an equal volume of 2 × SDS loading buffer and incubated for 10 min at 70-85°C, and were then analyzed by immunoblot analysis.

### 4.15 Statistical analysis

Statistical analysis was performed using GraphPad Prism software, and differences were analyzed using a Student’s *t-test*. Significance is denoted in the figures as follows: *, *P < 0.05*; **, *P < 0.01*; ***, *P < 0.001*; and ns, not significant.

## Data availability statement

The original contributions presented in the study are included in the article/**Supplementary Material**. Further inquiries can be directed to the corresponding author.

## Author contributions

SH, and W-hF conceived and designed experiments, analyzed data, and wrote the manuscript. XZ, YZ, YY, SL, and YX provided scientific insights and critical reagents. All authors contributed to the article and approved the submitted version.

## References

[1] Gaudreault NN Madden DW Wilson WC Trujillo JD Richt JA. African Swine fever virus: an emerging DNA arbovirus. Front Vet Sci (2020) 7:215. doi: 10.3389/fvets.2020.00215 32478103 PMC7237725

[2] Alonso C Borca M Dixon L Revilla Y Rodriguez F Escribano JM. ICTV virus taxonomy profile: asfarviridae. J Gen Virol (2018) 99(5):613–4. doi: 10.1099/jgv.0.001049 PMC12662184 29565243

[3] Xia NW Wang H Liu XL Shao Q Ao D Xu YL. African Swine fever virus structural protein p17 inhibits cell proliferation through ER stress-ROS mediated cell cycle arrest. Viruses (2021) 13(1):21. doi: 10.3390/v13010021 PMC7823474 33374251

[4] Alejo A Matamoros T Guerra M Andres G. A proteomic atlas of the African swine fever virus particle. J Virol (2018) 92(23):e01293–18. doi: 10.1128/JVI.01293-18 PMC6232493 30185597

[5] Karger A Perez-Nunez D Urquiza J Hinojar P Alonso C Freitas FB. An update on African swine fever virology. Viruses (2019) 11(9):864. doi: 10.3390/v11090864 31533244 PMC6784044

[6] Dixon LK Chapman DAG Netherton CL Upton C. African Swine fever virus replication and genomics. Virus Res (2013) 173(1):3–14. doi: 10.1016/j.virusres.2012.10.020 23142553

[7] He WR Yuan J Ma YH Zhao CY Yang ZY Zhang YH. Modulation of host antiviral innate immunity by African swine fever virus: a review. Animals (2022) 12(21):2935. doi: 10.3390/ani12212935 36359059 PMC9653632

[8] Bosch-Camos L Lopez E Rodriguez F. African Swine fever vaccines: a promising work still in progress. Porcine Health Manag (2020) 6:17. doi: 10.1186/s40813-020-00154-2 32626597 PMC7329361

[9] Kawai T Akira S. Toll-like receptors and their crosstalk with other innate receptors in infection and immunity. Immunity (2011) 34(5):637–50. doi: 10.1016/j.immuni.2011.05.006 21616434

[10] Sun LJ Wu JX Du FH Chen X Chen ZJJ. Cyclic GMP-AMP synthase is a cytosolic DNA sensor that activates the type I interferon pathway. Science (2013) 339(6121):786–91. doi: 10.1126/science.1232458 PMC3863629 23258413

[11] Luecke S Paludan SR. Molecular requirements for sensing of intracellular microbial nucleic acids by the innate immune system. Cytokine (2017) 98:4–14. doi: 10.1016/j.cyto.2016.10.003 27751656

[12] Dempsey A Bowie AG. Innate immune recognition of DNA: a recent history. Virology (2015) 479-480:146–52. doi: 10.1016/j.virol.2015.03.013 PMC4424081 25816762

[13] Akira S Uematsu S Takeuchi O. Pathogen recognition and innate immunity. Cell (2006) 124(4):783–801. doi: 10.1016/j.cell.2006.02.015 16497588

[14] Medzhitov R. Recognition of microorganisms and activation of the immune response. Nature (2007) 449(7164):819–26. doi: 10.1038/nature06246 17943118

[15] Tigano M Vargas DC Tremblay-Belzile S Fu Y Sfeir A. Nuclear sensing of breaks in mitochondrial DNA enhances immune surveillance. Nature (2021) 591(7850):477–81. doi: 10.1038/s41586-021-03269-w 33627873

[16] Carpenter S Ricci EP Mercier BC Moore MJ Fitzgerald KA. Post-transcriptional regulation of gene expression in innate immunity. Nat Rev Immunol (2014) 14(6):361–76. doi: 10.1038/nri3682 24854588

[17] Song JX Villagomes D Zhao HC Zhu M. cGAS in nucleus: the link between immune response and DNA damage repair. Front Immunol (2022) 13:1076784. doi: 10.3389/fimmu.2022.1076784 36591232 PMC9797516

[18] Chen SL Rong M Lv Y Zhu DY Xiang Y. Regulation of cGAS activity by RNA-modulated phase separation. EMBO Rep (2022) 24(2):e51800. doi: 10.15252/embr.202051800 36382803 PMC9900338

[19] Cao DF Han XA Fan XY Xu RM Zhang XZ. Structural basis for nucleosome-mediated inhibition of cGAS activity. Cell Res (2020) 30(12):1088–97. doi: 10.1038/s41422-020-00422-4 PMC7784699 33051594

[20] Shang GJ Zhang CG Chen ZJJ Bai XC Zhang XW. Cryo-EM structures of STING reveal its mechanism of activation by cyclic GMP-AMP. Nature (2019) 567(7748):389–93. doi: 10.1038/s41586-019-0998-5 PMC6859894 30842659

[21] Zhang CG Shang GJ Gui X Zhang XW Bai XC Chen ZJJ. Structural basis of STING binding with and phosphorylation by TBK1. Nature (2019) 567(7748):394–8. doi: 10.1038/s41586-019-1000-2 PMC6862768 30842653

[22] Ishikawa H Ma Z Barber GN. STING regulates intracellular DNA-mediated, type I interferon-dependent innate immunity. Nature (2009) 461(7265):788–92. doi: 10.1038/nature08476 PMC4664154 19776740

[23] Dobbs N Burnaevskiy N Chen DD Gonugunta VK Alto NM Yan N. STING activation by translocation from the ER is associated with infection and autoinflammatory disease. Cell Host Microbe (2015) 18(2):157–68. doi: 10.1016/j.chom.2015.07.001 PMC4537353 26235147

[24] Sharma S tenOever BR Grandvaux N Zhou GP Lin RT Hiscott J. Triggering the interferon antiviral response through an IKK-related pathway. Science (2003) 300(5622):1148–51. doi: 10.1126/science.1081315 12702806

[25] Song J Li K Li T Zhao G Zhou S Li H. Screening of PRRSV- and ASFV-encoded proteins involved in the inflammatory response using a porcine iGLuc reporter. J Virol Methods (2020) 285:113958. doi: 10.1016/j.jviromet.2020.113958 32827600

[26] Garcia-Belmonte R Perez-Nunez D Pittau M Richt JA Revilla Y. African Swine fever virus Armenia/07 virulent strain controls interferon beta production through the cGAS-STING pathway. J Virol (2019) 93(12):e02298–18. doi: 10.1128/JVI.02298-18 PMC6613762 30918080

[27] Li TT Zhao GH Zhang TQ Zhang ZX Chen X Song J. African Swine fever virus pE199L induces mitochondrial-dependent apoptosis. Viruses (2021) 13(11):2240. doi: 10.3390/v13112240 34835046 PMC8617669

[28] Woehnke E Fuchs W Hartmann L Blohm U Blome S Mettenleiter TC. Comparison of the proteomes of porcine macrophages and a stable porcine cell line after infection with African swine fever virus. Viruses (2021) 13(11):2198. doi: 10.3390/v13112198 34835004 PMC8620826

[29] Jia N Ou YW Pejsak Z Zhang YG Zhang J. Roles of African swine fever virus structural proteins in viral infection. J Vet Res (2017) 61(2):135–43. doi: 10.1515/jvetres-2017-0017 PMC5894393 29978065

[30] Civril F Deimling T Mann CCD Ablasser A Moldt M Witte G. Structural mechanism of cytosolic DNA sensing by cGAS. Nature (2013) 498(7454):332–7. doi: 10.1038/nature12305 PMC3768140 23722159

[31] Seo GJ Kim C Shin WJ Sklan EH Eoh H Jung JU. TRIM56-mediated monoubiquitination of cGAS for cytosolic DNA sensing. Nat Commun (2018) 9(1):613. doi: 10.1038/s41467-018-02936-3 29426904 PMC5807518

[32] Zhang X Wu JX Du FH Xu H Sun LJ Chen Z. The cytosolic DNA sensor cGAS forms an oligomeric complex with DNA and undergoes switch-like conformational changes in the activation loop. Cell Rep (2014) 6(3):421–30. doi: 10.1016/j.celrep.2014.01.003 PMC3969844 24462292

[33] Andreeva L Hiller B Kostrewa D Lassig C Mann CCD Drexler DJ. cGAS senses long and HMGB/TFAM-bound U-turn DNA by forming protein-DNA ladders. Nature (2017) 549(7672):394–8. doi: 10.1038/nature23890 28902841

[34] Fu YZ Guo Y Zou HM Su S Wang SY Yang Q. Human cytomegalovirus protein UL42 antagonizes cGAS/MITA-mediated innate antiviral response. PloS Pathog (2019) 15(5):e1007691. doi: 10.1371/journal.ppat.1007691 31107917 PMC6527189

[35] Fu YZ Su S Gao YQ Wang PP Huang ZF Hu MM. Human cytomegalovirus tegument protein UL82 inhibits STING-mediated signaling to evade antiviral immunity. Cell Host Microbe (2017) 21(2):231–43. doi: 10.1016/j.chom.2017.01.001 28132838

[36] Huang ZF Zou HM Liao BW Zhang HY Yang Y Fu YZ. Human cytomegalovirus protein UL31 inhibits DNA sensing of cGAS to mediate immune evasion. Cell Host Microbe (2018) 24(1):69–80.e4. doi: 10.1016/j.chom.2018.05.007 29937271

[37] Wu JX Sun LJ Chen X Du FH Shi HP Chen C. Cyclic GMP-AMP is an endogenous second messenger in innate immune signaling by cytosolic DNA. Science (2013) 339(6121):826–30. doi: 10.1126/science.1229963 PMC3855410 23258412

[38] Shi CR Yang XK Liu Y Li HP Chu HY Li GH. ZDHHC18 negatively regulates cGAS-mediated innate immunity through palmitoylation. EMBO J (2022) 41(11):e109272. doi: 10.15252/embj.2021109272 35438208 PMC9156970

[39] Ivashkiv LB Donlin LT. Regulation of type I interferon responses. Nat Rev Immunol (2014) 14(1):36–49. doi: 10.1038/nri3581 24362405 PMC4084561

[40] Hopfner KP Hornung V. Molecular mechanisms and cellular functions of cGAS-STING signalling. Nat Rev Mol Cell Biol (2020) 21(9):501–21. doi: 10.1038/s41580-020-0244-x 32424334

[41] Kato K Omura H Ishitani R Nureki O. Cyclic GMP-AMP as an endogenous second messenger in innate immune signaling by cytosolic DNA. Annu Rev Biochem (2017) 86:541–66. doi: 10.1146/annurev-biochem-061516-044813 28399655

[42] Ma Z Damania B. The cGAS-STING defense pathway and its counteraction by viruses. Cell Host Microbe (2016) 19(2):150–8. doi: 10.1016/j.chom.2016.01.010 PMC4755325 26867174

[43] Biolatti M Dell'Oste V Pautasso S Gugliesi F von Einem J Krapp C. Human cytomegalovirus tegument protein pp65 (pUL83) dampens type I interferon production by inactivating the DNA sensor cGAS without affecting STING. J Virol (2018) 92(6):e01774–17. doi: 10.1128/JVI.01774-17 PMC5827387 29263269

[44] Zhu ZX Li PF Yang F Cao WJ Zhang XL Dang W. Peste des petits ruminants virus nucleocapsid protein inhibits beta interferon production by interacting with IRF3 to block its activation. J Virol (2019) 93(16):e00362–19. doi: 10.1128/JVI.00362-19 PMC6675899 31167907

[45] Georgana I Sumner RP Towers GJ de Motes CM. Virulent poxviruses inhibit DNA sensing by preventing STING activation. J Virol (2018) 92(10):e02145–17. doi: 10.1128/JVI.02145-17 PMC5923072 29491158

[46] Razzuoli E Franzoni G Carta T Zinellu S Amadori M Modesto P. Modulation of type I interferon system by African swine fever virus. Pathogens (2020) 9(5):361. doi: 10.3390/pathogens9050361 32397378 PMC7281450

[47] Reis AL Netherton C Dixon LK. Unraveling the armor of a killer: evasion of host defenses by African swine fever virus. J Virol (2017) 91(6):e02338–16. doi: 10.1128/JVI.02338-16 PMC5331812 28031363

[48] Galindo I Alonso C. African Swine fever virus: a review. Viruses (2017) 9(5):103. doi: 10.3390/v9050103 28489063 PMC5454416

[49] Fraczyk M Wozniakowski G Kowalczyk A Bocian L Kozak E Niemczuk K. Evolution of African swine fever virus genes related to evasion of host immune response. Vet Microbiol (2016) 193:133–44. doi: 10.1016/j.vetmic.2016.08.018 27599940

[50] Sun MW Yu SX Ge HL Wang T Li YF Zhou PP. The A137R protein of African swine fever virus inhibits type I interferon production *via* the autophagy-mediated lysosomal degradation of TBK1. J Virol (2022) 96(9):e0195721. doi: 10.1128/jvi.01957-21 35412346 PMC9093111

[51] Dodantenna N Ranathunga L Chathuranga WAG Weerawardhana A Cha JW Subasinghe A. African Swine fever virus EP364R and C129R target cyclic GMP-AMP to inhibit the cGAS-STING signaling pathway. J Virol (2022) 96(15):e0102222. doi: 10.1128/jvi.01022-22 35861515 PMC9364804

[52] Wang XX Wu J Wu YT Chen HJ Zhang SF Li JX. Inhibition of cGAS-STING-TBK1 signaling pathway by DP96R of ASFV China 2018/1. Biochem Biophys Res Commun (2018) 506(3):437–43. doi: 10.1016/j.bbrc.2018.10.103 30348523

[53] Zhang JJ Zhao J Xu SM Li JH He SP Zeng Y. Species-specific deamidation of cGAS by herpes simplex virus UL37 protein facilitates viral replication. Cell Host Microbe (2018) 24(2):234–48.e5. doi: 10.1016/j.chom.2018.07.004 30092200 PMC6094942

[54] Su CH Zheng CF. Herpes simplex virus 1 abrogates the cGAS/STING-mediated cytosolic DNA-sensing pathway *via* its virion host shutoff protein, UL41. J Virol (2017) 91(6):e02414–16. doi: 10.1128/JVI.02414-16 PMC5331819 28077645

[55] Christensen MH Jensen SB Miettinen JJ Luecke S Prabakaran T Reinert LS. HSV-1 ICP27 targets the TBK1-activated STING signalsome to inhibit virus-induced type I IFN expression. EMBO J (2016) 35(13):1385–99. doi: 10.15252/embj.201593458 PMC4931188 27234299

[56] Wu JJ Li WW Shao YM Avey D Fu BS Gillen J. Inhibition of cGAS DNA sensing by a herpesvirus virion protein. Cell Host Microbe (2015) 18(3):333–44. doi: 10.1016/j.chom.2015.07.015 PMC4567405 26320998

[57] Zhang GG Chan B Samarina N Abere B Weidner-Glunde M Buch A. Cytoplasmic isoforms of kaposi sarcoma herpesvirus LANA recruit and antagonize the innate immune DNA sensor cGAS. Proc Natl Acad Sci USA (2016) 113(8):E1034–43. doi: 10.1073/pnas.1516812113 PMC4776510 26811480

[58] Zheng XJ Nie SM Feng WH. Regulation of antiviral immune response by African swine fever virus (ASFV). Virol Sin (2022) 37(2):157–67. doi: 10.1016/j.virs.2022.03.006 PMC9170969 35278697

[59] Yu L Liu PD. Cytosolic DNA sensing by cGAS: regulation, function, and human diseases. Signal Transduct Target Ther (2021) 6(1):170. doi: 10.1038/s41392-021-00554-y 33927185 PMC8085147

[60] O'Donnell V Holinka LG Krug PW Gladue DP Carlson J Sanford B. African Swine fever virus Georgia 2007 with a deletion of virulence-associated gene 9GL (B119L), when administered at low doses, leads to virus attenuation in swine and induces an effective protection against homologous challenge. J Virol (2015) 89(16):8556–66. doi: 10.1128/JVI.00969-15 PMC4524225 26063424

[61] O'Donnell V Risatti GR Holinka LG Krug PW Carlson J Velazquez-Salinas L. Simultaneous deletion of the 9GL and UK genes from the African swine fever virus Georgia 2007 isolate offers increased safety and protection against homologous challenge. J Virol (2017) 91(1):e01760–16. doi: 10.1128/JVI.01760-16 PMC5165186 27795430

[62] Borca MV Ramirez-Medina E Silva E Vuono E Rai A Pruitt S. Development of a highly effective African swine fever virus vaccine by deletion of the I177L gene results in sterile immunity against the current epidemic Eurasia strain. J Virol (2020) 94(7):e02017–19. doi: 10.1128/JVI.02017-19 PMC7081903 31969432

[63] Monteagudo PL Lacasta A Lopez E Bosch L Collado J Pina-Pedrero S. BA71DeltaCD2: a new recombinant live attenuated African swine fever virus with cross-protective capabilities. J Virol (2017) 91(21):e01058–17. doi: 10.1128/JVI.01058-17 PMC5640839 28814514

[64] Reis AL Goatley LC Jabbar T Sanchez-Cordon PJ Netherton CL Chapman DAG. Deletion of the African swine fever virus gene DP148R does not reduce virus replication in culture but reduces virus virulence in pigs and induces high levels of protection against challenge. J Virol (2017) 91(24):e01428–17. doi: 10.1128/JVI.01428-17 PMC5709585 28978700

[65] Sanchez-Cordon PJ Jabbar T Berrezaie M Chapman D Reis A Sastre P. Evaluation of protection induced by immunisation of domestic pigs with deletion mutant African swine fever virus BeninDeltaMGF by different doses and routes. Vaccine (2018) 36(5):707–15. doi: 10.1016/j.vaccine.2017.12.030 PMC5783716 29254837

[66] Chen WY Zhao DM He XJ Liu RQ Wang ZL Zhang XF. A seven-gene-deleted African swine fever virus is safe and effective as a live attenuated vaccine in pigs. Sci China Life Sci (2020) 63(5):623–34. doi: 10.1007/s11427-020-1657-9 PMC7223596 32124180

[67] Li D Wu P Liu H Feng T Yang W Ru Y. A QP509L/QP383R-deleted African swine fever virus is highly attenuated in swine but does not confer protection against parental virus challenge. J Virol (2022) 96(1):e0150021. doi: 10.1128/JVI.01500-21 34613824 PMC8754219

[68] Huang C Zhang Q Guo XK Yu ZB Xu AT Tang J. Porcine reproductive and respiratory syndrome virus nonstructural protein 4 antagonizes beta interferon expression by targeting the NF-kappa b essential modulator. J Virol (2014) 88(18):10934–45. doi: 10.1128/JVI.01396-14 PMC4178863 25008936

[69] Du L Liu YH Du YP Wang HL Zhang MJ Du YJ. Porcine reproductive and respiratory syndrome virus (PRRSV) up-regulates IL-15 through PKC beta 1-TAK1-NF-kappa b signaling pathway. Virology (2016) 496:166–74. doi: 10.1016/j.virol.2016.06.007 27318153

[70] Cao Y Qiu T Kathayat RS Azizi SA Thorne AK Ahn D. ABHD10 is an s-depalmitoylase affecting redox homeostasis through peroxiredoxin-5. Nat Chem Biol (2019) 15(12):1232–40. doi: 10.1038/s41589-019-0399-y PMC6871660 31740833

[71] Brigidi GS Bamji SX. Detection of protein palmitoylation in cultured hippocampal neurons by immunoprecipitation and acyl-biotin exchange (ABE). J Vis Exp (2013) 72):50031. doi: 10.3791/50031 PMC3605617 23438969

