# Supplementary material for: African swine fever virus QP383R dampens type I interferon production by promoting cGAS palmitoylation
Source: Front Immunol. 2023 May 9;14:1186916. doi: 10.3389/fimmu.2023.1186916 (PMC10203406; doi:10.3389/fimmu.2023.1186916)
Supplement: Supplementary file 1 [file DataSheet_1.docx]

**African swine fever virus (ASFV) QP383R dampens type I interferon production by promoting cGAS palmitoylation**

**Siyuan Hao^1,2,3,4^, Xiaojie Zheng^1,2,3,4^, Yingqi Zhu^1,2,3,4^, Yao Yao^1,2,3,4^, Sihan Li^1,2,3,4^, Yangyang Xu^1,2,3,4^, and Wen-hai Feng^1,2,3,4*^**

^1^State Key Laboratory of Agrobiotechnology, ^2^Frontiers Science Center for Molecular Design Breeding, ^3^Ministry of Agriculture Key Laboratory of Soil Microbiology, ^4^Department of Microbiology and Immunology, College of Biological Sciences, China Agricultural University, Beijing 100193, China

* **Correspondence:**

Wen-hai Feng DVM & PhD

College of Biological Science, China Agricultural University

Beijing 100193, China

Tel.: +86 10 62733335

Fax: +86 10 62732012

E-mail address: [whfeng@cau.edu.cn](mailto:whfeng@cau.edu.cn)


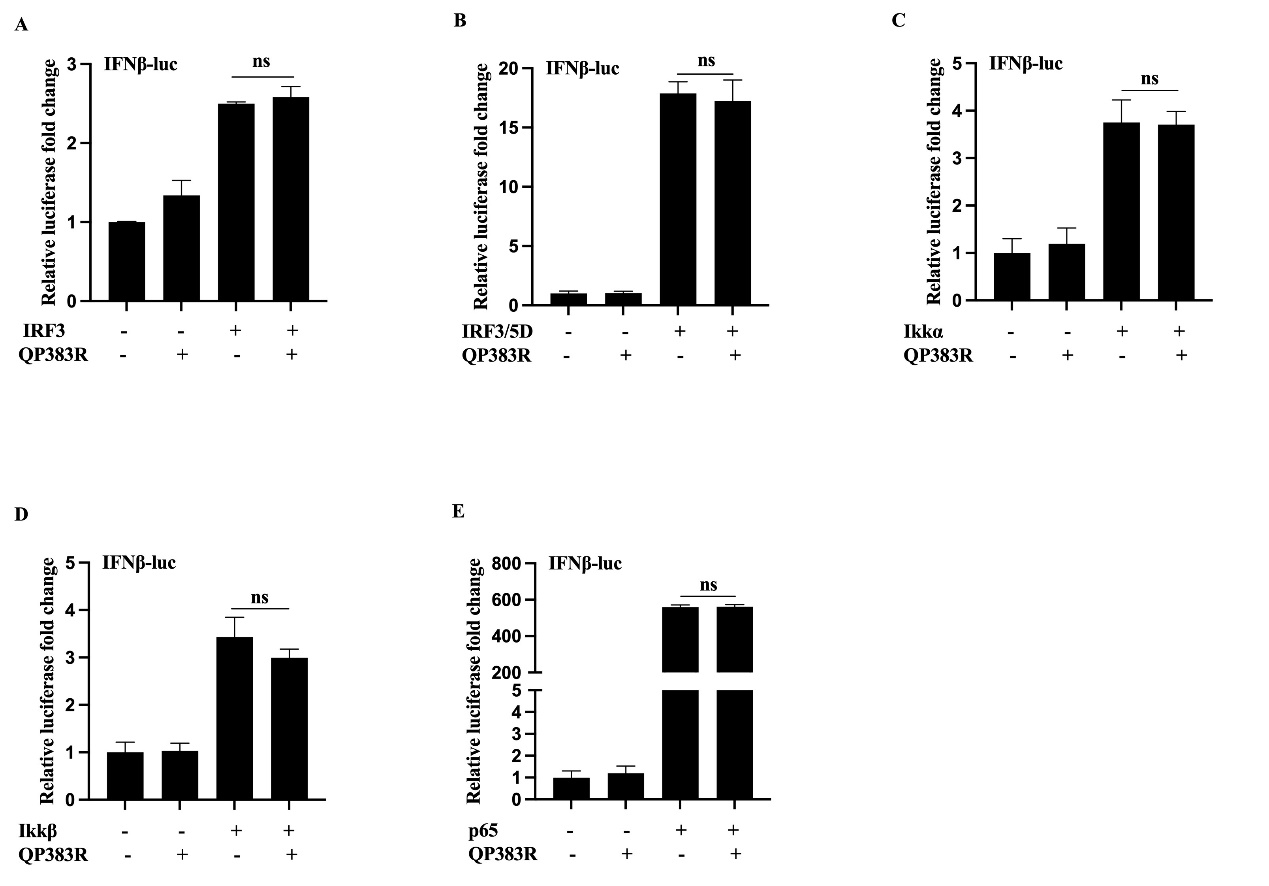


**Supplementary Figure 1. QP383R did not target steps downstream of STING in the cGAS-STING signal pathway.** (A-E) HEK239T cells were co-transfected with IFN-luc reporter promoter plasmid, pRL-TK, the expression plasmids for IRF3, IRF3/5D, Iκκα, Iκκβ or P65 along with QP383R or empty control plasmid. At 24 h post-transfection, cells were analyzed using dual-luciferase reporter assays. The data are representative of three independent experiments (means ± the standard errors of the mean [SEM]). ns, not significant (Student t test).

Table 1 Sequences of the primers used in real-time PCR

| **Name** | | **Forward Sequence (5’-3’)** | **Reverse Sequence (5’-3’)** |
| --- | --- | --- | --- |
| Human | IFNB1 | ACGCCGCATTGACCATCTAT | GTCTCATTCCAGCCAGTGCT |
|  | ISG54 | CTGCAACCATGAGTGAGAA | CCTTTGAGGTGCTTTAGATAG |
|  | ISG56 | TACAGCAACCATGAGTACAA | TCAGGTGTTTCACATAGGC |
|  | CXCL10 | GTGGCATTCAAGGAGTACCTC | GACCTTTCCTTGCTAACTGCT |
|  | GAPDH | CTGTTCGACAGTCAGCCGCATC | GCGCCCAATACGACCAAATCCG |
| Porcine | IFNB1 | AGCACTGGCTGGAATGAAAC | TCCAGGATTGTCTCCAGGTC |
|  | ISG15 | TGAAGATGCTGGGAGGCAAG | CACCCCATCCTGAAGCACAT |
|  | ISG54 | TCTGTGGCTTTGCACCTCTT | GGGGTTTCAGCTCCATTCCA |
|  | IL-6 | TGGATAAGCTGCAGTCACAG | ATTATCCGAATGGCCCTCAG |
|  | CXCL10 | TGCCCACATGTTGAGATCAT | CGGCCCATCCTTATCAGTAG |
|  | GAPDH | CCTTCCGTGTCCCTACTGCCAAC | GACGCCTGCTTCACCACCTTCT |

Table 2 Sequences of the primers used for amplificate plasmids

| **Name** | **Forward Sequence (5’-3’)** | **Reverse Sequence (5’-3’)** |
| --- | --- | --- |
| PRK5-HA-QP383R | CTGACTATGCGGGCGGATCCATGGCCTCCATTCTCACGCT | GGGCCATGGCGGCCAAGCTTTTAAGAAAAAGAAGAAGAGTGGCTC |
| PRK5-Flag-QP383R | GACGACGATGACAAGATGGCCTCCATTCTCACGCT | TGGGCCATGGCGGCCTTAAGAAAAAGAAGAAGAGTGGCTC |
| Myc-QP383R | GGCCATGGAGGCCCGAATTCATGGCCTCCATTCTCACGCT | CGCGGCCGCGGTACCTCGAGTTAAGAAAAAGAAGAAGAGTGGCTC |
| His-QP383R | GCTGATATCGGATCCATGGCCTCCATTCTC | GCGGCCGCAAGCTTTTAAGAAAAAGAAGAAGA |
| Flag-QP383R 1-31aa | GACGATGACAAGGGATCCATGGCCTCCATTCTC | GCCATGGCGGCCAAGCTTTTAAGGATTCTTGCCAGC |
| Flag-QP383R 32-283aa | GACGATGACAAGGGATCCATGCTAAGCTTTTATATTCAA | GCCATGGCGGCCAAGCTTTAAGGCATGTCTTTTGTAGATAT |
| Flag-QP383R 284-383aa | GACGATGACAAGGGATCCATGGTTGAAGGCCCGAAG | GCCATGGCGGCCAAGCTTTTAAGAAAAAGAAGAAGAGTG |
| Flag-QP383R 1-283aa | GACGATGACAAGGGATCCATGGCCTCCATTCTC | GCCATGGCGGCCAAGCTTTTAAGGCATGTCTTTTGTAGATAT |
| Flag-QP383R 32-383aa | GACGATGACAAGGGATCCATGCTAAGCTTTTATATTCAA | GCCATGGCGGCCAAGCTTTTAAGAAAAAGAAGAAGAGTG |
| Flag-cGAS RD | GACGATGACAAGGGATCCATGGCGGCCCGGCG | GCCATGGCGGCCAAGCTTTTAGGGGGGTGCCTCCATC |
| Flag-cGAS NTase | GACGATGACAAGGGATCCATGGGCGCCTGGAAGCTCCA | GCCATGGCGGCCAAGCTTTTACCAGCTGCTTTTAGATTCCAA |
| Flag-cGAS Mab21 | GACGATGACAAGGGATCCATGCCTGCTAGCACCCAGAAAG | GCCATGGCGGCCAAGCTTTCACCAAAAAACTGGAAATCCATTG |
| Flag-cGAS RD deletion | GACGATGACAAGGGATCCATGGGCGCCTGGAAGCTCCA | GCCATGGCGGCCAAGCTTTCACCAAAAAACTGGAAATCCATTG |
| HA-cGAS | CTGACTATGCGGGCGGATCCATGGCGGCCCGGCG | ACGCCGGGCCGCCATTCACCAAAAAACTGGAAATCCATTG |
| GST-cGAS | CCCTGGGATCCCCGGAATTCATGGCGGCCCGGCG | GCGCCGGGCCGCCATTCACCAAAAAACTGGAAATCCATTG |
